# Associations of screen time, sedentary time and physical activity with sleep in under 5s: A systematic review and meta-analysis

**DOI:** 10.1016/j.smrv.2019.101226

**Published:** 2020-02

**Authors:** Xanne Janssen, Anne Martin, Adrienne R. Hughes, Catherine M. Hill, Grigorios Kotronoulas, Kathryn R. Hesketh

**Affiliations:** a University of Strathclyde, School of Psychological Science and Health, Glasgow, UK; b University of Glasgow, MRC/CSO Social and Public Health Sciences Unit, Glasgow, UK; c School of Clinical Experimental Sciences, Faculty of Medicine, University of Southampton, UK; d Institute of Education, University College London, UK; e Southampton Children's Hospital, UK; f School of Medicine, Dentistry & Nursing, University of Glasgow, UK; g UKCRC Centre for Diet and Activity Research (CEDAR) at the MRC Epidemiology Unit, University of Cambridge School of Clinical Medicine, Institute of Metabolic Science, UK

**Keywords:** Infant, Toddler, Preschool, Sleep, Physical activity, Sedentary behavior, Screen time, PRISMA, Preferred Reporting Items for Systematic Reviews and Meta-Analyses, GRADE, Grading of Recommendations, Assessment, Development and Evaluation, RCT, Randomized controlled trial

## Abstract

Sleep is crucial to children's health and development. Reduced physical activity and increased screen time adversely impact older children's sleep, but little is known about these associations in children under 5 y. This systematic review examined the association between screen time/movement behaviors (sedentary behavior, physical activity) and sleep outcomes in infants (0–1 y); toddlers (1–2 y); and preschoolers (3–4 y). Evidence was selected according to Preferred Reporting Items for Systematic Reviews and Meta-Analyses guidelines and synthesized using vote counting based on the direction of association. Quality assessment and a Grading of Recommendations, Assessment, Development and Evaluation was performed, stratified according to child age, exposure and outcome measure. Thirty-one papers were included. Results indicate that screen time is associated with poorer sleep outcomes in infants, toddlers and preschoolers. Meta-analysis confirmed these unfavorable associations in infants and toddlers but not preschoolers. For movement behaviors results were mixed, though physical activity and outdoor play in particular were favorably associated with most sleep outcomes in toddlers and preschoolers. Overall, quality of evidence was very low, with strongest evidence for daily/evening screen time use in toddlers and preschoolers. Although high-quality experimental evidence is required, our findings should prompt parents, clinicians and educators to encourage sleep-promoting behaviors (e.g., less evening screen time) in the under 5s.

## Glossary of terms

*Total sleep duration*: Total time spent asleep over 24-hours (including naps if this was included in the original study). A longer total sleep duration was treated as favorable.
*Night awakenings*: Frequency of child waking up during the night. Less night awakenings were treated as favorable.
*Sleep onset latency*: Length of time for a child to transition from full wakefulness to sleep after “lights out”. Shorter transition time from full wakefulness to sleep after lights out was treated as favorable.
*Bedtime*: Time a child is put to bed. An earlier bed time was treated as favorable.
*Daytime napping*: Child naps during the day (yes/no). Child napping was treated as favorable.
*Sleep efficiency*: Percentage of total sleep duration spent asleep after sleep onset. A higher percentage of time spent asleep after sleep onset was treated as favorable.
*Sleep stability*: Score based on stable average sleep duration. More stable sleep duration was treated as favorable.
*Sleep quality*: Combination score based on different sleep outcomes (e.g., bed time, number of night awakenings, sleep onset latency). Better sleep quality was treated as favorable; this classification was driven by the reporting of included papers.

## Introduction

Adequate sleep plays a critical role in children's health and development, particularly in the early years. Short sleep duration in preschool children is linked to obesity in later childhood [1]. Furthermore, sleep problems beyond age two are associated with reduced grey matter volume at seven years, indicating a role of sleep in early brain development [2].

International guidelines recommend that infants (0–1 y) sleep for up to 17 h/d, while toddlers (1–3 y) and preschoolers (3–5 y) should sleep between 10 and 14 h/d [3]. However, today's children sleep less than they did a century ago [4] and 20–30% of parents report that their child has difficulties falling or staying asleep [5,6]. The causes for this apparent epidemic of sleep problems are likely multi-factorial but lifestyle changes in an increasingly digitized world are a cause for concern [7].

Australia, Canada, South Africa, New Zealand and WHO have issued 24-h movement guidelines for under 5s, recommending an ‘optimal day’ in terms of children's sleep, physical activity and sedentary behaviors (including screen time) [[8], [9], [10], [11]]. This ‘whole day matters’ approach places each behavior along a continuum, where declines in one behavior results in an increase in another. Studies in older children and adults have shown that daytime physical activity and screen time both influence sleep [[12], ∗[13], [14]], but less is known about these relationships in children under 5 y of age. The early years are also a critical period in life for establishing healthy behaviors as screen time and physical activity appear to track from early into later childhood and adolescence and consequently may influence sleep later in life [15].

No reviews to date have synthesized and evaluated the quality of international research evidence in the under 5s. This review therefore sought to determine how screen time, sedentary time and physical activity are associated with eight sleep outcomes (i.e., total sleep duration; night awakenings; sleep onset latency; bed time; daytime napping; sleep efficiency; sleep stability; and sleep quality) in children aged 0–4 y.

## Methods

### Data sources and search strategy

This systematic review was conducted and reported according to the Preferred Reporting Items for Systematic Reviews and Meta-Analyses (PRISMA) [16]. A systematic literature search was undertaken in April 2018 and updated in March 2019, using search terms related to: population; study design; outcome; exposure; and exclusion of clinical populations (Appendix 1). The search was conducted in 17 electronic databases: EBSCO (CINAHL); Cochrane Library (CENTRAL); OVID (EMBASE, MEDLINE, PsycINFO) and Web of Science (all databases). Citations were downloaded into Endnote citation management software (Thomson Reuters, Philadelphia, PA, USA) and de-duplicated. Included papers were searched for additional relevant publications, as were relevant reviews. No language or publication date restrictions were placed on the search.

### Study selection

Studies were included if they: 1) reported results from a cross-sectional, longitudinal or experimental study and 2) assessed the relationship between screen time (total daily screen time; evening screen time) or any movement behavior (i.e., sedentary time; total, light, moderate-to-vigorous physical activity; floor-based play (infants); outdoor play/time; sports participation) and any sleep outcomes reported. Studies assessed healthy children (i.e., general populations, including those with overweight/obesity) aged birth to 59 mo at baseline; objective or subjective measures of exposures and outcomes were considered. Exclusion criteria included: 1) clinical populations (e.g., children with chronic health conditions e.g., asthma, or developmental disorders e.g., cerebral palsy, autism) 2) qualitative studies; 3) studies assessing screen-based content; and 4) those assessing electromagnetic radiation.

### Study screening, data extraction and quality assessment

Identified titles and abstracts were screened for relevance (KH, GK) and included titles were separated by exposure type (sedentary time, physical activity or screen time; KH). Full texts of identified articles were retrieved and read in full to assess eligibility for inclusion (physical activity: KH, RK; sedentary behaviors: XJ, AM). Reviewers independently extracted and cross-checked relevant data using a pre-piloted data extraction form (physical activity: AM, KH; sedentary behaviors: AH, CH, XJ). Data were extracted per age group; infants (0 to <1 y), toddlers (1 to <3 y), preschoolers (3 to <5 y) and for each exposure-outcome association. The split between age groups was chosen for two reasons. First, major developmental differences exist during the early years in both physical and cognitive development. Therefore, we hypothesize that the investigated associations may be different for each of these age groups. Second, the chosen split in age groups is consistent with the international 24-h movement guidelines.

Investigated exposures were: 1) daily and evening screen time including parent report of child time spent on TV, tablet, phone, playing computer games, using the internet; 2) accelerometry measured physical activity including total sedentary behavior, light physical activity and moderate-to-vigorous physical activity; 3) parent reported floor based play, organized sport and outdoor play. Total sedentary behavior and screen time were treated as two different exposures to provide more detailed evidence about whether screen time or all sedentary behaviors influence sleep.

For longitudinal studies, all time points up to age five were included. The latest time point included was before, or as soon after, the children were five years old (>5 y if no follow-up data on <5 y was provided). If two or more papers reported on the same study sample, both were treated as separate studies if they reported different exposure-outcome relationships (n = 4) [[17], [18], [19], [20]]. Several papers examined multiple exposure-outcome associations (e.g., how total screen time and TV time were associated with sleep) and reported findings for different groups (e.g., examined differences across age groups, by time of the week, or by sex). Each exposure-outcome was therefore counted as an individual association, e.g., a paper examining the association between screen time and total sleep duration, but reporting results for weekdays and weekend days separately, was counted as one study but two associations. For experimental studies, differences in outcomes between control and intervention groups over time were used to assess influence of exposures. Where possible, results from adjusted multivariable models were reported.

Reviewers who extracted the data also assessed the methodological quality of primary studies and any discrepancies were resolved by consensus. Risk of bias assessment was completed as part of the Grading of Recommendations, Assessment, Development and Evaluation (GRADE) of evidence quality. Six domains (selection, performance, detection, attrition, reporting, and other sources of bias) specific to study design (observational or experimental) were assessed. Each domain was determined to have a low, unclear or high risk of bias [21].

### Data synthesis

Due to the heterogeneous nature of included studies, and the range of exposures and outcomes assessed, meta-analysis was only appropriate for one exposure-outcome association total screen time and sleep duration in infants, toddlers and preschoolers. Where available, correlation coefficients were recorded for each study. If studies did not report correlations coefficients but provided beta coefficients these were converted in to correlation coefficients using the method described by Peterson and Brown (2005). Only studies reporting cross-sectional associations were included in the main analysis. A sensitivity analysis including longitudinal outcomes was conducted but no significant differences were found between the two models. Data were pooled in a random-effect meta-analyses using Comprehensive Meta-analysis, version 3.3.07. Heterogeneity across studies was assessed using I^2^ statistics (I^2^ of 0–40% represents low heterogeneity and 75–100% considerable heterogeneity) [23].

For the remaining associations, as recommended by the Cochrane handbook for systematic reviews of interventions, vote counting based on the direction of association was conducted [24], comparing the number of favorable to unfavorable associations. Favorable associations were categorized as those where the exposure measure resulted in a positive association with sleep outcomes (e.g., less screen time associated with longer total sleep duration). Associations were unfavorable if the exposure measure resulted in a negative association with sleep (e.g., more screen time associated with shorter total sleep duration). Summary results per exposure-outcome association were presented as number of unfavorable (for screen time and sedentary behavior) and favorable (for physical activity, outdoor play and sport club attendance) associations divided by the total number of studies included. A binomial probability test was conducted. The p-value from this test indicates the probability of observing the summary results if the exposure-outcome associations were in the opposite direction, thus a small p-value indicates a higher probability the results are valid [24]. This method does not rely on p-values reported by the authors of the primary studies.

GRADE was performed on all findings, stratified according to child age (infants; toddlers; preschoolers), exposure, and outcome measure, with possible range from very low to high [25,26]. GRADE assigns an initial rating to each study design (i.e., high for randomized controlled trials, low for observational studies - both longitudinal and cross-sectional). This was then upgraded or downgraded according to the risk of bias, inconsistency, indirectness, imprecision, publication bias, dose–response relationship, residual confounding or the size of the magnitude.

## Results

### Characteristics of identified articles

Initial searches identified 1604 articles, 90 full-text articles were screened, and of these, 31 studies (29 unique cohorts) met the inclusion criteria and were included in the data analysis (Fig. S1). A total of 60,445 children were included, ranging from 22 [27] to 39,813 [28] participants per article (age range 0–4.99 y). Included articles were published between 2007 and 2019 and conducted in North America (n = 10) [17,19,27,28,[30], [31], [32], [33], [34], [35]]; Europe (n = 7) [[36], [37], [38], [39], [40], [41], [42]]; Asia (n = 8) [29,[43], [44], [45], [46], [47], [48], [49]]; Australasia (n = 5) [19,20,[50], [51], [52]]; and one article included participants from multiple countries [53].

One article reported an experimental design (RCT; [36]), seven were longitudinal [19,30,38,41,47,50,51] (of which four also analyzed data cross-sectionally [30,47,50,51]) and 23 were cross-sectional [17,18,20,[27], [28], [29],[31], [32], [33], [34], [35],^37^,^39^,^40^,[42], [43], [44], [45], [46],^48^,^49^,^52^,^53^]. Eleven articles examined the association between physical activity and sleep [19,20,31,33,36,40,44,45,49,51,52], five articles examine the association between sedentary time and sleep [20,27,31,36,49] and 23 articles examined the association between screen time and sleep [17,18,[28], [29], [30],^32^,^34^,^35^,[37], [38], [39], [40], [41], [42], [43],[45], [46], [47], [48],[50], [51], [52], [53]]. Eight articles had an age range covering more than one age group (infants and toddlers n = 4 [18,30,43,47]; toddlers and preschoolers n = 2 [41,51]; infants, toddlers and preschoolers n = 1 [17]; Table 1).

**Table 1:** Study characteristics.

Study author and year	Type of study	Country	Sample	Age	Age group	Exposure	Exposure description	Sleep outcomes	Findings	Covariates included in analysis
Ahn et al., 2016 [43]	Cross-sectional	Korea	N = 1033	Age range: 0–36 mo	Infants and toddlers	Evening screen time	Parent reported television or video	Sleep duration, bedtime; night awakenings	TV at sleep initiation was associated with a later bedtime (β = 0.30). TV at sleep initiation was not significantly associated with any of the other sleep outcomes (direction of association not reported).	child demographic variables (age, sex, birth order), parental demographic variables (age, educational level, employment status), parental behaviors at bedtime, and other aspects of the sleep ecology (sleep arrangement, location, position)
Cespedes et al., 2014 [17]	Cross-sectional	USA	N = 6 mo: 1673; 1 y: 1227; 2 y: 1360; 3 y: 1242; 4 y: 1202	Age range: 6 mo - 4 y	Infants, toddlers and preschoolers	Total daily screen time	Parent reported television	Sleep duration	Higher TV time was associated with shorter sleep duration at ages 6 mo (β = −3.0; 95% CI, −8.0 to 2.0); 1 (β = −6.0; 95% CI, −9.0 to −2.0), 2 (β = −6.0; 95% CI, −10.0 to −2.0); 3 (β = −2.0; 95% CI, −6.0 to −2.0); and 4 (β = −4.0; 95% CI, −8.0 to 0.0) y.	child age in years at time of assessment, race/ethnicity, gender, maternal education, and household income; age 4 analysis additionally adjusted for TV in bedroom.
Chonchaiya et al., 2017 [30]	Longitudinal and cross-sectional	USA	N = 208	Mean age: 6.2 mo (time 1); 12.3 mo (time 2)	Infants and toddlers	Total daily screen time, evening screen time	Parent reported use of all electronic media	Sleep duration; sleep latency; night awakenings; naptime duration	Higher levels of total daily screen time at age 12 mo was associated with longer sleep latency at age 12 mo (β = 0.16 for weekday; β = 0.17 for weekend day). Total daily screen time at 6 mo was associated with longer sleep latency at 6 mo (during weekends). Total and evening screen time for 6 and 12 mo of age was not significantly associated with any other sleep outcomes at age 12 mo (direction of association not reported). Bedroom media use at 12 mo was not significantly associated with sleep latency at 12 mo (direction of association not reported).	age, gender, co-sleeping status, evening media use at age 12 mo, maternal education, and household income were included in the final regression models as covariates.
De Bock et al., 2013 [36]	RCT	Germany	N = 809	Mean age (SD): 5.05 y (0.2) Age range: 4–6 y	Preschoolers	Total PA, SB, MVPA	Accelerometry	Sleep quality	A trend toward improved subjective sleep quality in the intervention group was noted (β = −0.113; 95% CI, −0.003 to 0.23).	Intention-to-treat basis. The core model assumed a linear change of the outcomes with time and included two normally distributed random effects (one at the preschool level and one at the child level) to adjust for clustering in the data due to the hierarchic sampling scheme. Further, all models included the variables age, gender, rural versus urban community of preschools, and season as covariates to adjust for a potential confounding effect of these variables.
Duraccio et al., 2017 [31]	Cross-sectional	USA	N = 131	Mean age (SD): 4.9 y (0.5)	Preschoolers	SB, MVPA, VPA	Accelerometry	Sleep duration	For each added day of high sedentary behavior (i.e., being in top tertile of sedentary behavior), the probability of obtaining sufficient sleep decreased (1 d = 0.56; 95% CI, 0.26–0.75; 2 d = 0.51; 95% CI, 0.37–0.65; 3 d = 0.22; 95% CI, 0.11–0.33). MVPA and VPA were not associated with sleep duration.	Interaction with sex tested, ns
Garrison et al., 2011 [32]	Cross-sectional	USA	N = 617	Mean age (SD): 51 mo (8)	Preschoolers	Total daily screen time, evening screen time	Parent reported use of all electronic media	Sleep quality	Total screen time was associated with higher sleep problem scores (β = 0.244; 95%CI, 0.113 to 0.375). Each additional hour of evening screen time was associated with increases in sleep problem scores (β = 0.743; 95% CI, 0.373 to 1.114). Each additional hour of day screen time was associated with increases in sleep problem scores (β = 0.107; 95% CI, −0.047 to 0.260).	child gender, low-income status, single-adult household, and SCBE (Social Competence and Behavior Evaluation) internalizing and externalizing scores, as well as which parent completed the survey (mother versus other), each additional hour of nonviolent daytime media time, and each additional hour of violent daytime media time
Genuneit et al., 2018 [37]	Cross-sectional	Germany	N = 530	Approximate age: 3 y	Preschoolers	Total daily screen time	Parent reported use of all electronic media, TV/DVD, computer/internet use, computer gaming	Sleep habits	Total daily screen time, TV/DVD time, computer/internet use and computer gaming were associated with inconsistent sleep habits.	NA
Hager et al., 2016 [33]	Cross-sectional	USA	N = 240	Mean age: 20.2 mo	Toddlers	MVPA	Accelerometry	Sleep duration, Sleep quality	MVPA was associated with longer sleep duration (β = 0.332; SE, 0.138). Those with high sleep behavior scores (5–6) spent significantly more time in MVPA (65.3 min) compared to those with mid-range sleep behavior scores (3–4; 45.3 min) but not those with low scores (0–2; 58.3 min). Those with high sleep behavior scores (5–6) had significantly higher counts per minute (433.1 cpm) compared to those with mid-range sleep behavior scores (3–4; 348.8 cpm) but not those with low scores (0–2; 409.2 cpm).	NA
Hauck et al., 2018 [27]	Cross-sectional	USA	N = 22	Approximate age: 6mo ± 1 wk	Infants	SB	Sedentary behavior and screen time	Sleep duration; night awakenings; daytime napping	More time in SB was significantly associated with less total sleep (r = −0.524) and non-significantly associated with less night time sleep (r = −0.417), more time awake at night (r = 0.308), reduced nap duration (r = −0.104), reduced nap frequency (r = −0.068)	NA
Ikeda et al., 2012 [29]	Cross-sectional	Japan	N = 39,813	Approximate age: 4.5 y	Preschoolers	Total daily screen time	Parent reported television; computer games	Sleep duration, daytime napping	Those watching more hours of television were more likely to have shorter (<10hr) sleep durations (OR not playing = 1.0; <1hr = 1.01; 95% CI, 0.67 to 1.52; 1–2hr = 1.06; 95% CI, 0.71 to 1.58; 2–3hr = 1.37; 95% CI, 0.92 to 2.04; 3–4hr = 1.55; 95% CI, 1.04 to 2.33; ≥4hr = 1.91; 95% CI, 1.26, 2.90). Playing computer games was unfavorably associated with sleep duration (not playing = 1; <1hr = 1.11; 95% CI, 1.02 to 1.21; 1–2hr = 1.14; 95% CI, 0.98 to 1.32; ≥2hr = 1.62; 95% CI, 1.18 to 2.23. Playing computer games was associated with less daytime napping (not playing OR = 1; <1hr = 0.85; 95% CI, 0.78 to 0.92; 1–2hr = 0.80; 95% CI, 0.69 to 0.92; ≥2hr = 0.57; 95% CI, 0.40 to 0.82 Television time was associated with more daytime napping (not watching = 1; <1hr = 1.07; 95% CI 0.75 to 1.53; 1–2hr = 1.15; 95% CI, 0.81 to 1.64; 2–3hr = 1.16; 95% CI 0.82 to 1.65; 3–4hr = 1.21; 95% CI, 0.85–1.72; ≥4hr = 1.22; 95% CI, 0.85 to 1.77	regional population, gender, existence of older siblings, years of maternal and paternal education, hours spent watching, television, hours spent playing computer games, paternal and maternal work hours, and whether or not the child attended preschool, or a childcare center
Iwata et al., 2011 [44]	Cross-sectional	Japan	N = 48	Approximate age: 5 y	Preschoolers	Sports participation	Parent reported sports participation	Sleep onset; sleep end time; sleep latency; sleep efficiency	Sport lesson attendance was associated with earlier sleep onset on weekdays (B = −0.258; 95% CI, −0.728 to 0.043) and later onset on weekends (B = 0.096; 95% CI, −0.391 to 0.760). Sport lesson attendance was associated with earlier sleep end on weekdays (B = −0.342; 95% CI, −0.641 to −0.062) but not weekends (B = 0.086; 95% CI, −0.331 to 0.598). Sport less attendance was associated with longer sleep latency (B = 0.318; 95% CI, 0.393, 7.149 for weekdays; B = 0.307; 95% CI, 0.245 to 6.921 for weekends). Sport less attendance was associated with higher sleep efficiency (B = 0.318; 95% CI, 0.393 to 7.149)	NA
Ji et al., 2018 [45]	Cross-sectional	China	N = 112	Age range: 3–6 y	Preschoolers	MVPA; screen time	Accelerometry; parent reported electronic media use	Sleep duration	Those engaging in more MVPA (OR = 0.735; 95%CI, 0.189 to 2.855) or daily screen time (OR = 0.380; 95%CI, 0.107 to 1.348) were less likely to get sufficient sleep (8–13hr).	Age, gender, father's BMI, mother's BMI, total physical activity time, daily steps and daily sedentary time.
Krejci et al., 2011 [46]	Cross-sectional	Czech Republic and Japan	N = 1096; Czech Republic: 497; Japan: 599	Mean age: Czech Republic: 4.6 y (1.1); Japan: 3.8 y (1.2)	Preschoolers	Total daily screen time	Parented reported use of computer games	Sleep duration; bedtime	Frequency of playing computer games was not associated with sleep duration but was associated with later bedtime. Duration of playing computer games was not associated with sleep duration but was associated with later bedtime in Czech children but not Japanese. Time of the day of playing computer games was associated with shorter sleep duration bedtime in Czech children but not Japanese.	NA
Magee et al., 2014 [50]	Longitudinal and cross-sectional	Australia	N = 3427	Age range: 4–5 y (time 1); 6–7 y (time 2)	Preschoolers	Total daily screen time	Parent reported television and video use; computer use; total screen time	Sleep duration	Total screen time at age 4 was associated with shorter sleep duration at age 6 (B = −0.06; 95% CI, −0.10 to −0.02) TV/video viewing at age 4 was associated with shorter sleep duration at age 6 (B = −0.05; 95% CI, −0.09 to −0.01). Computer use at age 4 was unfavorably associated with sleep duration at age 6 (B = −0.10; 95% CI, −0.21 to 0.01). Total screen time at age 4 was associated with shorter sleep duration at age 4 (B = −0.10; 95% CI not reported).	child's sex, baseline obesity status, sleep problems, household income, and maternal education
Marinelli et al., 2014 [38]	Cross-sectional and longitudinal	Spain	N = 1202 (time 1); 1090 (time 2)	Approximate age: Time 1: 2 y, Time 2: 4 y	Toddlers	Total daily screen time	Parent reported television time	Sleep duration	Children with longer periods of television viewing at age 2 (≥1.5 h per day) had shorter sleep duration and each additional hour of television viewing decreased sleep duration (β = −0.13; 95% CI, −0.19 to −0.08). Children with longer periods of television viewing at age 2 (≥1.5 h per day) had shorter sleep duration at age 4 and each additional hour of television viewing decreased sleep duration (β = −0.11; 95% CI, −0.18 to −0.05).	BMI at baseline, BMI change, parental educational level, sex
McDonald et al., 2014 [39]	Cross-sectional	United Kingdom	N = 1702	Mean age: 15.8 mo	Toddlers	Evening screen time	Parent reported television time	Sleep duration	Children with more >1hr morning television viewing had an increased risk of short sleep duration (<11hr; OR = 1.13; 95% CI, 0.80 to 1.58). Children with more >1hr evening television viewing had an increased risk of short sleep duration (<11hr; OR = 1.89; 95% CI, 1.26 to 2.84).	Maternal education, ethnicity, sex, birth weight, older children around, evening TV, age, daytime sleep, regular night waking.
Mindell et al., 2013 [53]	Cross-sectional	Australia, New Zealand, Canada, United Kingdom, United States, China, Hong Kong, India, Japan, South Korea, Malaysia, Philippines, Singapore, Thailand	N = 2590; Australia and New Zealand: 286; Canada: 272; United Kingdom: 298; United States: 284; China: 248; Hong Kong: 82; India: 294; Japan: 48; South Korea: 312; Malaysia: 121; Philippines: 76; Singapore: 81; Thailand: 88)	Age range: 3–6 y	Preschoolers	Total daily screen time;	Parent reported television, computer or electronic game use	Sleep duration; sleep latency; bedtime; night awakenings;	More screen time was associated with longer sleep latency (r = 0.11), later bedtime (r = 0.21), more night awakenings (r = 0.07) and longer night time sleep duration (r = 0.08). More screen time was not significantly associated with duration of night awakenings, total sleep duration, or daytime sleep (direction of association not reported).	NA
Nathanson et al., 2018 [34]	Cross-sectional	USA	N = 402	Age range: 3–5 y	Preschoolers	Total daily screen time; evening screen time	Parent reported television use; mobile electronic device use	Sleep duration	More time spent using a tablet during the evening (β = 0.12; SE, 0.12), smartphone (β = 0.03; SE, 0.20), game player (β = 0.06; SE, 0.20), iPod or watching TV (β = 0.2; SE, 0.07) were associated with lower sleep duration. More time spent using a tablet (β = 0.13; SE, 0.04), iPod (β = 0.02; SE, 0.10) or watching TV (β = 0.2; SE, 0.03) was associated with lower sleep duration. More time spent using a smartphone (β = −0.1; SE, 0.07), or laptop (β = −0.01; SE, 0.06) was associated with longer sleep duration.	Mothers education, mothers employment, household income, child's age, childcare attendance, TV viewing,
Nathanson et al., 2014 [35]	Cross-sectional	USA	N = 107	Mean age (SD): 53.4 mo (0.87)	Preschoolers	Total daily screen time; evening screen time	Parent reported television time	Sleep duration	More time spent watching TV during the evening was associated with shorter sleep duration (r = −0.3). Background TV time all day, background TV time in the daytime and background TV time in the night time was correlated with shorter sleep duration (r = −0.3, r = −0.3; r = −0.2, respectively). Total time spent watching TV or time spent watching TV during the day were correlated with sleep duration (r = −0.2; r = −0.1, respectively).	Income education and age
Nevarez et al., 2010 [18]	Cross-sectional	USA	N = 1676 (time 1); 1228 (time 2); 1365 (time 3)	Approximate age: 6 mo (time 1); 12 mo (time 2); 24 mo (time 3)	Infants, Toddlers	Total daily screen time	Parent reported television time	Sleep duration	At age 6 mo' more time spent watching TV was associated with shorter sleep duration (β = −0.1; 95%CI, −0.16 to 0.02). At age 12 mo more time spent watching TV was associated with shorter sleep duration (β = −0.1; 95%CI, −0.18 to −0.04). At age 24 mo more time spent watching TV was associated with shorter sleep duration (β = −0.1, 95%CI: −0.15 to −0.02).	Maternal age, parents education, household income, sex, race/ethnicity
Ota et al., 2007 [48]	Cross-sectional	Not reported	N = 330	Mean age (SD): 4.2 y	Preschoolers	Total daily screen time	Parent reported television time	Sleeping habits	Those in the regular sleeping habits group watched significantly less TV than those in the irregular group (1.7hr/d ± 1.1 compared to 2.0 h/d ± 1.2).	NA
Plancoulaine et al., 2015 [40]	Cross-sectional	France	N = 1028	Approximate age: 3 y	Preschoolers	Outside PA; Total daily screen time	Parent reported television time and other screens	Sleep duration	More time spent watching TV was associated with shorter sleep duration (<12hr/d) in boys (OR = 1.65; 95% CI, 1.23 to 2.21) but not girls (OR = 1.06; 0.76 to 1.47). Outside physical activity was not associated with sleep duration.	Socio-economic factors; Family income; Educational level; Childcare arrangements; Maternal isolation/depression (for girls); Maternal BMI; night waking (for girls); parent present at falling asleep; watching TV; Food score; BMI z-score
Reynaud et al., 2016 [41]	Longitudinal	France	N = 1346	Approximate age: 2 y (time 1); 3 y (time 2); 5–6 y (time 3)	Toddlers and Preschoolers	Total daily screen time	Parent reported television time	Night awakenings	Those spending more time watching TV at age 3 y were more likely to belong to the 2–5 common night awakenings trajectory at age 5–6 y (OR = 1.3; 95% CI, 1.13 to 1.58).	Childcare center, Parental education status, Household income, Maternal depression, Child gender, Child ponderal index, First child, Passive smoking at home, Collective care arrangement, Atopic profile, Ear nose throat infection, Falling asleep with parental presence, Bottle feeding at night, Activity, Shyness, Emotionality.
Séguin et al., 2016 [28]	Cross-sectional	Canada	N = 52	Approximate age = 45 mo	Preschoolers	Total daily screen time	Parent reported television time, computer, game console or other electronics use	Sleep patterns	More time using the computer (r = −0.38), video game console use (r = −0.32) and other electronic media use (r = −0.33) was associated with shorter sleep duration.	NA
Sijtsma et al., 2015 [42]	Cross-sectional	The Netherlands	N = 759	Age range: 3–4 ys	Preschoolers	Total daily screen time	Parent reported television time	Sleep duration	Higher amounts of screen time were associated with shorter sleep duration (r = −0.16).	NA
Taylor et al., 2015 [19]	Longitudinal	New Zealand	N = 143	Mean age (SD): 3.0 y (0.0)	Preschoolers	MVPA; total PA	Accelerometry	Sleep stability	Children displaying a more stable sleep pattern had higher levels of day-time physical activity than all other groups (Mean (SD) MVPA: low average 97 (47) minutes; variable medium sleep 91 (39) minutes; high average sleep 79 (35) minutes; consistent medium sleep 111 (49) minutes). Children displaying a more stable sleep pattern had higher counts per minutes than all other groups (Mean (SD) CPM: low average 791 (266) minutes; variable medium sleep 790 (234) minutes; high average sleep 725 (208) minutes; consistent medium sleep 913 (332) minutes).	NA
Vijakkhana et al., 2015 [47]	Longitudinal and cross-sectional	Thailand	N = 208	Approximate age: 6 mo (time 1); 12 mo (time 2)	Infants and Toddlers	Total daily screen time; evening screen time	Parent reported screen media use	Sleep duration	Evening media exposure at 6mo was associated with shorter 6mo night time sleep duration (weekday r = −0.3; weekend day r = −0.2). Evening media exposure at 12mo was associated with shorter 12mo night time sleep duration (r = −0.2 for both weekday and weekend day). Evening media exposure at 6mo was associated with shorter 12mo night time sleep duration (r = −0.2 for both weekday and weekend day). Higher levels of media viewing at 6mo was associated with shorter 6mo night time sleep during weekdays (r = −0.1) but not during weekends (r = 0.0) Higher levels of media viewing at 12mo were not associated with 12mo night time sleep duration (r = 0.0 for both weekday and weekend). Higher levels of media viewing at 6mo were not associated with 12mo night time sleep duration (r = 0.0 for both weekday and weekend).	12-mo bedroom media use, chronological age at 12mo, gender, 12mo cosleeping status, maternal education in y, mothers and fathers income in Baht
Wang et al., 2019 [49]	Cross-sectional	Taiwan	N = 183	Average age (SD): 6.61 mo (0.36)	Infants	Total PA; SB; floor play	Accelerometry and parent reported floor play	Sleep duration; sleep efficiency	PA was significant associated with a lower sleep percentage (β = −0.02), and non-significantly associated with less 24-h sleep (β = −0.03) and more time napping (β = 0.03) SB was significantly associated with less total 24-h sleep (β = −5.89) and not significantly associated with higher sleep percentage (β = 0.06) and more time napping (β = 1.41). Floor play was associated with less total 24-h sleep (β = −4.18), higher sleep percentage (β = 0.14) and less time napping (β = −3.56) but none were significant.	Gender, infant BMI, breastfeeding status, maternal employment status
Williams et al., 2014 [20]	Cross-sectional	New Zealand	N = 216	Approximate age: 3 y (time 1); 5 y (time 2)	Preschoolers	Total PA, SB, LPA, MVPA	Accelerometry	Sleep duration; night awakenings	The most active children spent 0.92 h (55 min) less time asleep at night compared with the least active children at 3 years of age. More active children were also awake more at night, for 16–19 min. These children spent less time in sedentary activity (2.49 h at age 3) and more time in light (0.14 h) and MVPA (2.95 h).	Awake at night; Sedentary time; Light activity; MVPA
Xu H et al., 2016 [51]	Longitudinal and cross-sectional	Australia	N = 497 (time 1); 415 (time 2); 369 (time 3)	Approximate age: 2 y (time 1); 3.5 years (time 2); 5 y (time 3)	Toddlers and Preschoolers	Outdoor play; Total daily screen time	Parent reported electronic media use	Sleep duration; bedtime; sleep latency; night awakenings	Higher levels of screen time at age 2 were associated with shorter night time sleep (β = −0.1; 95% CI, −0.23 to −0.03) and longer sleep latency (β = −2.5; 95% CI, 0.63 to −4.35) at age 2. Those with higher levels of screen time at age 2 were less likely to be in the long sleep group (>10hr/d; OR = 0.8; 95% CI, 0.64 to 0.95) and more likely to wake up at night (OR = 1.4; 95% CI, 1.15 to 1.72) at age 2. Levels of screen time at age 3.5 y were not associated with night time sleep (β = 0.0; 95% CI, −0.09 to 0.05). Higher levels of screen time at age 3.5 y were associated with longer sleep latency at age 3.5 y (β = 0.4; 95% CI, −1.16 to 1.97). Those with higher levels of screen time at age 3.5 y were not more likely to be in the long sleeping group or wake up at night at age 3.5 y (OR = 1; 95% CI 0.86–1.18; OR = 1.0; 95% CI, 0.82 to 1.10, for sleep duration and night awakenings respectively). Higher levels of screen time at age 2 y were associated with shorter night time sleep (β = −0.1; 95% CI, −0.09 to −0.01) and longer sleep latency (β = 1.6; 95% CI, 0.53 to 2.63) at age 5 y. Those with higher levels of screen time at age 2 y were less likely to be in the long sleeping group at age 5 (OR = 0.87; 95% CI, 0.76 to 1.0) and were more likely to wake up at night at age 5 y (OR = 1.53; 95% CI, 1.10 to 2.14).	childcare attendance, annual household income, mother's country of birth, age, education level, employment and marital status at baseline.
Zhang et al., 2019 [52]	Cross-sectional	Australia	N = 173	Average age: 19.7 mo	Toddlers	Total PA; MVPA; total daily screen time	Accelerometry and parent reported screen time	Sleep duration; sleep quality; sleep variability	Those participating in <302.9 min/d TPA had increased chances of sleeping > 646.8 min/d (OR = 2.38; 95%CI, 1.27–4.45), being in the high variability (>59.2 min/d difference between days) group (OR = 1.27; 95%CI, 0.68–2.40) and sleep problems (OR = 1.33; 95%CI, 0.71–2.50) Those participating in <55.1 min/d MVPA had an increased chance of sleeping > 646.8 min/d (OR = 1.06; 95%CI, 0.85–1.95), of being in the high variability (>59.2 min/d difference between days) group (OR = 1.23; 95%CI, 0.66–2.31), and had less chance of experiencing sleep problems (OR = 0.96; 95%CI, 0.51–1.79) Those who did not meet the screen time guidelines had a lower chance of sleeping > 646.8 min/d (OR = 0.98; 95%CI, 0.38–2.51), a greater chance of being in the high variability (>59.2 min/d difference between days) group (OR = 2.13; 95%CI, 0.77–5.90) and a greater chance of experiencing sleep problems (OR = 1.41; 95%CI, 0.55–3.65)	Age, sex, socio-economic status, body mass index

PA, physical activity; SB, sedentary behavior; MVPA, moderate-to-vigorous physical activity; VPA, vigorous physical activity; OR, Odds ratio; β, adjusted beta coefficient; B, unadjusted beta coefficient; r, Pearsons correlation coefficient; SE, standard error; 95% CI, 95% confidence interval.

### Total daily screen time

The relationship between total daily screen time and sleep was examined in 20 studies (infants n = 4; toddlers n = 9; preschoolers n = 16; Table 2) [17,18,[28], [29], [30],^32^,^34^,^35^,^37^,^38^,[40], [41], [42],[46], [47], [48],[50], [51], [52], [53]]. In infants, higher levels of total daily screen time were associated with shorter total sleep duration (3/5 associations), more night awakenings (1/1) and longer sleep onset latency (1/1). Toddlers' and preschoolers' total daily screen time was unfavorably associated with sleep outcomes in 39/43 associations. In toddlers, higher levels of total daily screen time were associated with shorter total sleep duration (7/8), more night awakenings and later bedtime (2/2), longer sleep onset latency (3/3), lower sleep quality (1/1) and worse sleep stability (1/1). In preschoolers, higher levels of total daily screen time were associated with shorter total sleep duration (12/13), more night awakenings (2/3), later bedtime and lower sleep quality (3/3), longer sleep onset latency (2/2), and less daytime napping (1/2). Few studies reported favorable associations between higher levels of total screen time and any sleep outcomes (n = 6 associations).

**Table 2:** Exposure: Screen time (TV, Tablet, Phone, Playing computer games, Using the internet).

Outcome	Age group	Unfavorably related to exposure	Favorably related to exposure	Summary		N participants (total (if n = 1) or range)	Quality
				n/N (%)*	P value^#^		
Sleep duration	Infants	[17,18,47] weekday	[47] weekend day^b^	3/5 (60.0)	0.375	Longitudinal: 208 Cross-sectional: 208 to 1676	Very low^a^
Night awakenings	Infants	[43]		1/1 (100.0)	0.500	Cross-sectional: 1033	Very low^a^
Sleep latency	Infants	[30]		1/1 (100.0)	0.500	Cross-sectional: 208	Very low^a^
Sleep duration	Toddlers	[17,18], [38]^b^, [47] weekend day [51]^b^, [52],	[47] weekday	7/8 (87.5)	0.035	Longitudinal: 369 to 1202 Cross-sectional: 173 to 1676	Very low^a^
Night awakenings	Toddlers	[51]^b^		2/2 (100.0)	0.250	Longitudinal: 369 Cross-sectional: 497	Very low^a^
Bedtime	Toddlers	[51]^b^		2/2 (100.0)	0.250	Longitudinal: 369 Cross-sectional: 497	Very low^a^
Sleep latency	Toddlers	[30,51,51]		3/3 (100.0)	0.125	Longitudinal: 369 Cross-sectional: 208 to 497	Very low^a^
Sleep quality	Toddlers	[52]		1/1 (100.0)		Cross-sectional: 173	Very low^a^
Sleep stability	Toddlers	[52]		1/1 (100.0)		Cross-sectional: 173	Very low^a^
Sleep duration	Preschoolers	[17,28,29,34] TV viewing, tablet use, iPod use [35,40,42,45,50,50,51,53],	[34] Smartphone, laptop	12/13 (92.3)	0.002	Longitudinal: 3427 Cross-sectional: 52 to 39,813	Very low^a^
Night awakenings	Preschoolers	[41]^b^, [53]	[51]	2/3 (66.6)	0.500	Longitudinal: 1346 Cross-sectional: 415 to 2590	Very low^a^
Bedtime	Preschoolers	[46] Computer use [51,53],		3/3 (100.0)	0.125	Cross-sectional: 415 to 2590	Very low^a^
Sleep latency	Preschoolers	[51,53]		2/2 (100.0)	0.250	Cross-sectional: 415 to 2590	Very low^a^
Sleep quality	Preschoolers	[32,37,48]		3/3 (100.0)	0.125	Cross-sectional: 330 to 617	Very low^a^
Daytime napping	Preschoolers	[29] TV time	[28] Computer games	1/2 (50.0)	0.750	Cross-sectional: 39,813	Very low^a^

*n = number of associations showing unfavorable association, N = total number of associations for the specific exposure-outcome relationship reported; ^#^two-sided p-value from the binomial probability test. Small p-value indicates higher probability that the results are valid.

a Quality of evidence was downgraded due to serious risk of bias. Quality rating of individual studies can be found in Table S1.

b Indicates Longitudinal study.

**Fig. 1:**
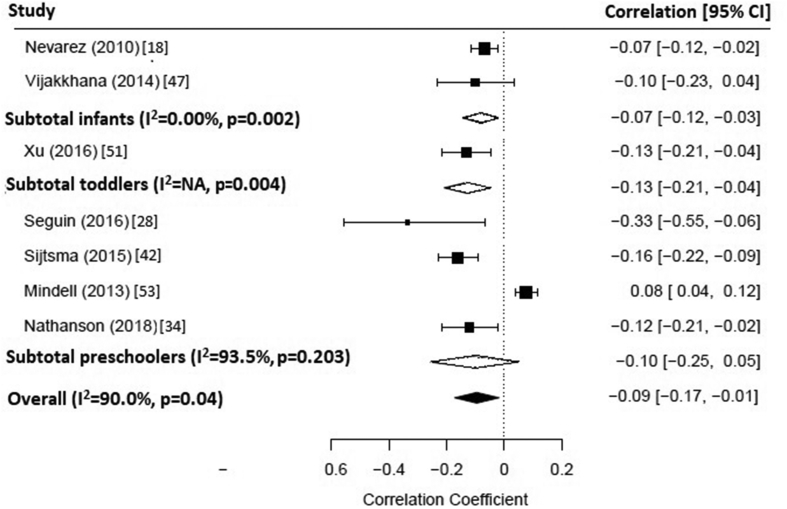
Forest plot of the effect of total screen time on sleep duration. CI, confidence interval.

A subset of seven studies were included in the random-effect meta-analysis to quantify the effect of total screen time on sleep duration (Fig. 1) [18,28,34,42,47,51,53]. The pooled correlation coefficient was −0.09 (95% CI: −0.17, −0.01; I^2^ = 90.0%; p = 0.04). Sub-group analysis showed similar results for infants (r = −0.07; 95% CI: −0.12, −0.03; I^2^ = 0.00%; p = 0.002) and toddlers (r = −0.13; 95% CI: −0.21, −0.04; I^2^ = NA; p = 0.004). However, in preschoolers the effect of total screen time on sleep duration became non-significant (r = −0.10; 95% CI: −0.25, 0.05; I^2^ = 93.5%; p = 0.203).

### Evening screen time

The relationship between evening screen time and sleep was examined in eight studies (infants n = 3; toddlers n = 4; preschool-aged children n = 4; Table 3) [30,22,34,35,39,43,46,47]. In infants, higher levels of evening screen time were associated with shorter nighttime sleep duration (2/2) and later bedtime (1/1). Toddlers' and preschoolers' evening screen time was unfavorably associated with sleep in 8/9 associations. In toddlers, higher levels of evening screen time were associated with shorter total sleep duration (2/2) and later bedtime (1/1). In preschoolers, higher levels of evening screen time were associated with shorter total sleep duration (3/4), later bedtime and lower sleep quality (1/1). Importantly, only one study reported a favorable association between evening screen time and any sleep outcome.

**Table 3:** Exposure: Evening screen time.

Outcome	Age group	Unfavorably related to exposure	Favorably related to exposure	Summary		N participants (total (if n = 1) or range)	Quality
				n/N (%)*	P value^#^		
Sleep duration	Infants	[47]^b^		2/2 (100.0)	0.250	Longitudinal: 208 Cross-sectional: 208	Very low^a^
Bedtime	Infants	[43]		1/1 (100.0)	0.500	Cross-sectional: 1033	Very low^a^
Sleep duration	Toddlers	[39,47]		2/2 (100.0)	0.250	Cross-sectional: 208 to 1702	Very low^a^
Bedtime	Toddlers	[43]		1/1 (100.0)	0.500	Cross-sectional: 1033	Very low^a^
Sleep duration	Preschoolers	[34] TV viewing, tablet, game player, iPod use [35,46],	[34] Smartphone, laptop	3/4 (75.0)	0.313	Cross-sectional: 107 to 1096	Very low^a^
Bedtime	Preschoolers	[46]		1/1 (100.0)	0.500	Cross-sectional: 1096	Very low^a^
Sleep quality	Preschoolers	[32]		1/1 (100.0)	0.500	Cross-sectional: 617	Very low^a^

*n = number of associations showing unfavorable association, N = total number of associations for the specific exposure-outcome relationship reported; ^#^two-sided p-value from the binomial probability test. Small p-value indicates higher probability that the results are valid.

a Quality of evidence was downgraded due to serious risk of bias. Quality rating of individual studies can be found in Table S1.

b IndicatesLongitudinal study.

### Total sedentary time

The association between total sedentary time and sleep was examined in five studies (infants n = 2; toddlers n = 0; preschoolers n = 3; Table 4) [20,27,31,36,49]. In infants, higher levels of total sedentary time were associated with shorter sleep time duration (2/2), more night awakenings (1/1), less daytime napping (1/2) and better sleep efficiency (1/1). In preschoolers, higher levels of total sedentary time were associated with shorter sleep time duration (1/2) associations and later bedtime (1/1). More sedentary time was associated with fewer night awakenings (1/1). A decrease in sedentary time showed an association with improved sleep quality (1/1). No evidence was available for toddlers.

**Table 4:** Total sedentary time and physical activity.

Outcome	Age group	Unfavorably related to exposure	Favorably related to exposure	Summary		N participants (total (if n = 1) or range)	Quality
				n/N (%)*	P value^#^		
**Exposure: sedentary behavior**							
Sleep duration	Infants	[27,49]		2/2 (100.0)	0.250	Cross-sectional: 22 to 183	Very low^a^
Night awakenings	Infants	[27]		1/1 (100.0)	0.500	Cross-sectional: 22	Very low^a^
Daytime napping	Infants	[27]	[49]	1/2 (100.0)	0.750	Cross-sectional: 22 to 183	Very low^a^
Sleep efficiency	Infants		[49]	1/1 (100.0)	0.500	Cross-sectional: 183	Very low^a^
Sleep duration	Preschoolers	[31]	[21]	1/2 (50.0)	0.750	Cross-sectional: 131 to 216	Very low^a^
Night awakenings	Preschoolers		[21]	0/1 (0.0)	0.500	Cross-sectional: 216	Very low^a^
Bedtime	Preschoolers	[20]		1/1 (100.0)	0.500	Cross-sectional: 216	Very low^a^
Sleep quality	Preschoolers	[36]^c^		1/1 (100.0)	0.500	RCT: 809	Moderate^a^
**Exposure: Total Physical Activity**							
Sleep duration	Infants	[49]		0/1 (0.0)	0.500	Cross-sectional: 183	Very low^a^
Sleep efficiency	Infants	[49]		0/1 (0.0)	0.500	Cross-sectional: 183	Very low^a^
Daytime napping	Infants	[49]		0/1 (0.0)	0.500	Cross-sectional: 183	Very low^a^
Sleep duration	Toddlers	[52]	[33]	1/2 (50.0)	0.750	Cross-sectional: 173 to 240	Very low^a^
Sleep quality	Toddlers		[33,52]	2/2 (100.0)	0.250	Cross-sectional: 173 to 240	Very low^a^
Sleep stability	Toddlers		[52]	1/1 (100.0)	0.500	Cross-sectional: 173 to 183	Very low^a^
Sleep duration	Preschoolers	[20]		0/1 (0.0)	0.500	Cross-sectional: 216	Very low^a^
Night awakenings	Preschoolers	[20]		0/1 (0.0)	0.500	Cross-sectional: 216	Very low^a^
Sleep stability	Preschoolers		[19]^b^	1/1 (100.0)	0.500	Cross-sectional: 143	Low
**Exposure: Light Physical Activity**							
Bedtime	Preschoolers	[20]		0/1 (0.0)	0.500	Cross-sectional: 216	Very low^a^
**Exposure: Moderate-to-vigorous physical activity**							
Sleep duration	Toddlers	[52]		0/1 (0.0)	0.500	Cross-sectional: 173	Very low^a^
Sleep quality	Toddlers	[52]	[33]	1/2 (50.0)	0.750	Cross-sectional: 173 to 240	Very low^a^
Sleep stability	Toddlers		[52]	1/1 (100.0)	0.500	Cross-sectional: 173	Very low^a^
Sleep duration	Preschoolers	[45]	[31]	1/2 (50.0)	0.750	Cross-sectional: 112 to 131	Low
Bedtime	Preschoolers	[20]		0/1 (0.0)	0.500	Cross-sectional: 216	Very low^a^
Sleep quality	Preschoolers		[36]	1/1 (100.0)	0.500	RCT: 809	Moderate^a^
Sleep stability	Preschoolers		[19]	1/1 (100.0)	0.500	Longitudinal: 143	Low
**Exposure: Floor-based play**							
Sleep duration	Infants	[49]		0/1 (0.0)	0.500	Cross-sectional: 183	Very low^a^
Sleep efficiency	Infants		[49]	1/1 (100.0)	0.500	Cross-sectional: 183	Very low^a^
Daytime napping	Infants	[46]		0/1 (0.0)	0.500	Cross-sectional: 183	Very low^a^
**Exposure: Outdoor play/outdoor time**							
Sleep duration	Toddlers	[51,51]		0/2 (100.0)	0.250	Longitudinal: 369 Cross-sectional: 497	Very low^a^
Night awakenings	Toddlers		[51]^b^	2/2 (100.0)	0.250	Longitudinal: 369 Cross-sectional: 497	Very low^a^
Bedtime	Toddlers		[51]^b^	2/2 (100.0)	0.250	Longitudinal: 369 Cross-sectional: 497	Very low^a^
Sleep latency	Toddlers		[51]^b^	2/2 (100.0)	0.250	Longitudinal: 369 Cross-sectional: 497	Very low^a^
Sleep duration	Preschoolers	[51]	[51]	1/2 (50.0)	0.750	Cross-sectional: 415 to 1028	Very low^a^
Night awakenings	Preschoolers		[51]	1/1 (100.0)	0.500	Cross-sectional: 415	Very low^a^
Bedtime	Preschoolers		[51]	1/1 (100.0)	0.500	Cross-sectional: 415	Very low^a^
Sleep latency	Preschoolers		[51]	1/1 (100.0)	0.500	Cross-sectional: 415	Very low^a^
**Exposure: Organized sport participation**							
Bedtime	Preschoolers		[44]	1/1 (100.0)	0.500	Cross-sectional: 48	Very low^a^
Sleep efficiency	Preschoolers		[44]	1/1 (100.0)	0.500	Cross-sectional: 48	Very low^a^

RCT: randomized controlled trial. *n = number of associations showing unfavorable association (sedentary behavior) or favorable association, N = total number of associations for the specific exposure-outcome relationship reported; #two-sided p-value from the binomial probability test. Small p-value indicates higher probability that the results are valid.

a Quality of evidence was downgraded due to serious risk of bias. Quality rating of individual studies can be found in Table S1.

b IndicatesLongitudinal study.

c Indicates RCT.

### Physical activity

The association between physical activity related behaviors (i.e., total physical activity, moderate-to-vigorous physical activity, outdoor play and sports participation) and sleep was examined in 11 studies (infants n = 1; toddlers n = 3; preschoolers n = 7; Table 4) [19,20,31,33,36,40,44,45,49,51,52]. The relationship between total physical activity and sleep was examined in five studies [19,20,33,49,52]. In infants, higher levels of total physical activity were associated with shorter total sleep duration, worse sleep efficiency and less daytime napping (1/1). In toddlers, higher levels of total physical activity were associated with longer total sleep duration (1/2), better sleep quality (2/2) and better sleep stability (1/1). In preschoolers, higher levels of total physical activity were associated with shorter total sleep duration and more night awakenings (1/1) and better sleep stability (1/1).

Seven studies assessed the relationship between physical activity intensity and sleep [19,20,31,33,36,45,52]. In one study, conducted in preschoolers, light physical activity was associated with later bedtime (1/1 association). In toddlers, higher levels of moderate-to-vigorous physical activity were associated with better sleep quality (1/2), and better sleep stability (1/1), and shorter total sleep duration (1/1). In preschoolers, higher levels of moderate-to-vigorous physical activity were associated with shorter total sleep duration (1/2) and later bedtime (1/1), better sleep quality and better sleep stability (1/1). No evidence was available for infants.

The relationship between floor-based play was examined in one study for infants [49]: floor-based play was associated with shorter total sleep duration, less daytime napping (1/1) and better sleep efficiency (1/1). The relationship between time spent playing outdoors and sleep was examined in two studies [40,51]. Toddlers' outdoor play was associated with shorter total sleep duration (2/2), shorter sleep onset latency, fewer night awakenings and earlier bedtime (2/2). Preschoolers' outdoor play was associated with longer total sleep duration (1/2) and fewer night awakenings, earlier bedtime and shorter sleep onset latency (1/1). Preschoolers' attendance at sports clubs was associated with earlier bedtime and better sleep efficiency (i.e., higher fraction of total sleep spent asleep after sleep onset; 1/1) [44].

### Quality of evidence

The quality of evidence ranged from very low to moderate for moderate-to-vigorous physical activity; and very low for all other exposure-outcome associations across age groups (Table 2, Table 3, Table 4). Most studies were downgraded due to a serious risk of bias (commonly due to use of exposure or outcome measures with unknown psychometric properties; Table S1).

## Discussion

To our knowledge, this is the first systematic review to explore how screen time and movement behaviors are associated with sleep in children under 5 y. This review highlighted a trend for an unfavorable association between higher levels of total daily and evening screen time and sleep outcomes in infants, toddlers and preschoolers; very few studies showed favorable screen-sleep associations. Meta-analysis conducted in a sub-sample of studies to examine the association between daily screen time and sleep duration confirmed these unfavorable associations in infants and toddlers. In preschoolers, the meta-analysis did not show a significant association, but this may be due to the heterogeneity of the included studies. Evidence for associations between total daily sedentary time/physical activity and sleep was less conclusive: there was an indication that more outdoor play and higher levels of moderate-to-vigorous physical activity were favorably associated with sleep outcomes in toddlers and preschoolers. Most evidence was from observational studies (both cross-sectional and longitudinal), did not show significant associations, and did not report on dose–response relationships leading to evidence frequently being classified as low quality. In addition, no clear differences were found between studies including large (>500) or small (<500) sample sizes.

Established early in childhood [41], sleep patterns are governed by a complex interplay of physiological, genetic, psychological and social/environmental factors. A range of behaviors, including physical activity, sedentary and screen time, may delay or displace sleep - ‘5 more minutes please!’ Moreover, socio-environmental influences such as parenting style, the home environment, and socioeconomic status are likely to influence young children's sleep, screen and activity behaviors [[54], [55], [56]].

In line with a recent systematic review in older children (5–20 y) [13], our review and meta-analysis highlights that screen time appears to be unfavorably associated with young children's sleep. Short wavelength (blue/green) light emitted from screens suppresses pineal melatonin secretion, influencing both circadian entrainment (via supra-chiasmatic nucleus signaling) and sleep onset (via the hypothalamic ventrolateral pre-optic nucleus) [57,58]. Although evidence is limited in very young children, differential diurnal rate of melatonin secretion appears to emerge early in development at around 27–41 d of age [59]. Theoretically therefore, evening screen exposure in very young children may not only delay sleep onset on exposure [60] but also potentially cause longer term disturbance to sleep stability [61]. A dim light environment prior to bedtime is likely to be conducive to melatonin secretion, simultaneously promoting earlier sleep onset whilst helping to establish and maintain an optimal circadian rhythm [62], with less night waking [63]. In addition to the light emitted from screens, the content, its interactivity, and subsequent level of arousal, may also adversely affect sleep.

Despite a wealth of evidence for a positive association between physical activity and sleep in older age groups [64], very few studies have examined the association between physical activity and total sedentary time on sleep in children 0–4 y. Our review identified evidence suggesting that more outdoor play and time spent engaged in moderate-to-vigorous physical activity may be associated with better sleep outcomes in toddlers and preschoolers. Although experimental research is largely lacking in children and young people, it has been noted in infants that natural light exposure, particularly during the afternoon, may improve nighttime sleep [65]. Such exposure, as part of children's outdoor play, may help to regulate melatonin secretion and circadian rhythm, encouraging regular sleep onset. Several other physiological mechanisms have also been proposed to explain how higher intensity physical activity may positively influence sleep (albeit in the context of adult sleep). These include: 1) activity triggering an increase in body temperature and subsequent cooling with rest to promote sleep onset, and 2) activity reducing negative arousal states which may otherwise lead to sleep problems [63]. Future experimental studies should determine why and how screen time and movement-behaviors impair and promote sleep respectively. This is particularly important given a number of 24-h movement behavior guidelines have recently been published worldwide, which outline an ‘optimal day’ for children's sleep, physical activity and sedentary behaviors (including screen time) [[8], [9], [10], [11]]. Where the ‘whole day matters’ and each behavior is placed along a continuum, declines in one behavior may feasibly result in an increase in another.

This review highlights important gaps in the evidence base around screen-based and movement behaviors, and sleep outcomes in young children. The quality of evidence summarized in this review was low and in some instances inconclusive. The variation in results may be due to the wide range of exposure and outcome measures used across studies. Moreover, study quality tended to be downgraded due to use of measurement tools with untested psychometric properties, with 20 out of the 28 articles reporting exposures measured using an unpublished questionnaire/failing to report the questionnaire's psychometric properties (Table S1). Studies included in this review frequently focused on television-based screen time, and did not examine the use of more contemporary screens (e.g., tablets, phones) and/or the type of activities children engaged in while using screens (e.g., watching a movie or talking to grandparents on tablet/phone). With the advances in technology over the last decade, it is important that studies now consider the influence of alternative electronic media and screen-based activities (such as e-readers and tablets) on children's sleep. In addition, it is important studies examine the influence different media content may have on children's sleep (e.g., education v. recreational content) [66] While screen-based technology can positively support learning [67], neglecting its influence on sleep may paradoxically constrain neurodevelopment in the under 5s.

While objective measures of sleep duration (e.g., accelerometry) and valid and reliable sleep questionnaires are available, very few studies used either to assess sleep outcomes here (n = 10). Accelerometry is known to poorly differentiate between prolonged sedentary behavior and sleep [68]; included studies using accelerometry all used different methods to estimate sleep and wake periods [18,19,31,36,45] which could have led to the discrepancy in results [69]. Standardized measurement and analysis procedures of exposure and outcomes would allow consistency and validity across studies. There was also a lack of experimental or intervention studies aiming to improve sleep practices in the early years. Last, as the majority of studies included in this review were cross-sectional, cause and effect could not be established. Thus, it is important to consider a possible reverse pathway, i.e., poor sleepers are more fatigued, resulting in more daily sedentary time and less physical activity. This review therefore highlights evidence gaps including the need to develop and evaluate interventions to improve sleep in young children, especially by reducing screen use before bedtime.

### Strengths and limitations

We applied rigorous review methods, including duplicate assessment at every stage. Given that this review was restricted to published studies, publication bias cannot however be ruled out. All included studies were conducted in high and middle-income countries. Almost half included small sample sizes (15 out of 31 studies had fewer than 500 participants), which may have limited their statistical power to detect significant associations. By using vote counting based on the direction of the effect, we limited the impact underpowered studies may have on the summarized results [24]. Nine exposures and nine outcome measures were used here, thus limiting the use of meta-analysis: where common exposure-outcome associations existed (i.e., for screen time and total sleep duration) meta-analysis was conducted. We defined the absence of daytime napping as an unfavorable outcome here as the majority of studies examining this outcome were in infants (n = 3 out of 4). However, daytime napping has been linked to adverse sleep outcomes such as irregular sleep habits in preschoolers [48]. It is therefore difficult to interpret whether napping is an un/favorable behavior when assessed as an isolated outcome. Due to the co-dependence of movement and sleep behaviors, an increase in one behavior would be expected to result in a decline in another, e.g., if physical activity leads to an increase in sleep duration, sedentary behavior is more than likely to decrease. Future studies would benefit from assessing co-dependent behaviors across a 24-h period. Last, most studies included in this review controlled for common confounders (e.g., age, socio-economic status, sex) but few controlled for characteristics in the home and wider environment which may impact sleep (e.g., chaotic home life, shared bedrooms, noise). Future research should consider a wider range of relevant confounders in order to fully elucidate the relationship between screen time, movement behaviors and young children's sleep.

## Conclusions

Screen time is unfavorably associated with multiple sleep outcomes in infants, toddlers and preschoolers. Conversely, in toddlers and preschoolers more time spent in outdoor play, and in higher intensity physical activity, was associated with better sleep outcomes. There is a pressing need for future research to establish how contemporary screen time (e.g., tablets and e-readers) influences the 24-h equipoise of activity and sleep in young children. Public health initiatives and policies are needed to help parents and educators encourage balanced use of screen-based technologies and positive movement behaviors to promote healthy lifestyles and development in the under 5s.

**Practice points**

1. While global and national 24-h movement guidelines suggest that a relationship between screen exposure and sleep exists, the recommendations do not explicitly address the relationship between sleep outcomes and other movement behaviors found here. Future 24-h movement behavior guidelines should therefore consider the findings from this review, which highlight the importance of limiting screen time, especially before bedtime, and providing sufficient exposure to natural daylight, in young children.
2. In a time when electronic media use among young children is becoming the norm, it is crucial to raise public (and particularly parental) awareness about the potential harmful effects exposure to screens may have on a young child's sleep and development.
3. Public health initiatives and policies that stress the importance limiting screen time before bed and the potential benefits of active outdoor play for sleep are warranted. For example, the American Academy of Pediatrics in the USA and the Royal College of Paediatrics and Child Health in the UK recently published guidelines recommending an hour's screen curfew before bedtime in children (https://pediatrics.aappublications.org/content/138/5/e20162591; https://www.rcpch.ac.uk/resources/health-impacts-screen-time-guide-clinicians-parents).

**Research agenda**

This review highlights important gaps in the evidence base around screen-based and movement behaviors, and sleep outcomes in young children.

1. Evidence of the validity and reliability of a broader range of screen time measures is needed, with papers here tending to focus on television-based screen time. In addition, standardized measurement and analysis procedures of exposure and outcomes would allow consistency and validity across studies.
2. It is important that studies start to consider the influence of alternative electronic media and screen-based activities (such as e-readers and tablets) on children's sleep.
3. Very few studies have examined the association between physical activity and total sedentary time on sleep in children 0–4 y. No evidence was available for the association between movement and screen behaviors and several of the sleep outcomes in certain age groups.
4. No studies were identified from lower-middle and low-income countries. These countries are likely to have substantial difference in the home and wider environment, which could influence both exposure and outcomes.

## Conflicts of interest

The authors do not have any conflicts of interest to disclose.
