# Supplementary material for: Associations of screen time, sedentary time and physical activity with sleep in under 5s: A systematic review and meta-analysis
Source: Sleep Med Rev. 2020 Feb;49:101226. doi: 10.1016/j.smrv.2019.101226 (PMC7034412; doi:10.1016/j.smrv.2019.101226)
Supplement: Multimedia component 1 [file mmc1.docx]

**Supplementary Online Content**

Janssen X, Martin A, Hughes AR, et al. Associations of Screen Time, Sedentary Time and Physical Activity with Sleep in Under 5s: A Systematic Review

**Appendix 1.** Search strategy

**Appendix Figure 1.** Flowchart outlining identification of papers for inclusion

**Appendix Table 1.** Quality assessment individual studies

This supplementary material has been provided by the authors to give readers additional information about their work.

**Appendix 1. Search strategy**

| Outcome | 1 | Sleep/ |
| --- | --- | --- |
|  | 2 | (sleep adj3 duration).tw. |
|  | 3 | (sleep adj onset adj latency).tw. |
|  | 4 | (wakefulness or awakening or parasomnia* or insomnia*).tw. |
|  | 5 | (sleep routine or sleep timing or sleep hygiene or healthy sleep habits or bedtime routine or sleep practice or bedtime practice or sleep environment or sleep schedule or time in bed or sleep onset latency).tw,kw |
|  | 6 | exp *Sleep Apnea Syndromes/ and (apnea or apnoea).ti. |
|  | 7 | *(1 or 2 or 3 or 4 or 5) not 6* |
|  | 8 | Polysomnography/ |
|  | 9 | Accelerometer/ or Accelerometry/ |
|  | 10 | Actigraphy/ |
|  | 11 | (polysomnogr* or actigr* or accelerom*).tw. |
|  | 12 | ((objectiv* adj3 measur*) or (direct* adj3 measure*)).tw. |
|  | 13 | (Sleep* adj3 (report or questionnaire* or index or eval* or diary or diaries or log* or journal*)).tw. |
|  | 14 | (self report* or proxy report*).tw. |
|  | 15 | *or/8-14* |
|  | 16 | *7 and 15* |
| Population | 17 | ("infant (1 to 23 months)" or "preschool child (2 to 5 years)") |
|  | 18 | (pre-school* or preschool* or early childhood).tw,kf. |
|  | 19 | (Toddler* or Nurser* or Baby or Babies or Kindergarten*).tw,kf. |
|  | 20 | *or/17-19* |
|  | 21 | *16 and 20* |
|  | 22 | *limit 21 to (journal article or published erratum or "retraction of publication")* |
|  | 23 | *limit 22 to (english)* |
| Screen | 24 | Television/ or Video Games/ or Software/ or Videodisc Recording/ or Cartoons as Topic/ or Motion Pictures as Topic/ |
|  | 25 | exp Internet/ or exp Computers, handheld/ |
|  | 26 | Communications Media/ or Mass Media/ |
|  | 27 | (television or screentime or ((screen or computer) adj3 time) or ((watch* or view*) adj2 (dvd* or video*)) or screen media or social media or video gam* or videogam* or computer gam* or electronic gam*).tw,kw. |
|  | 28 | (Smartphone* or ipad or apps or app or mobile applications).tw,kw. |
|  | 29 | screen based entertainment.tw,kw. |
|  | 30 | (Mobile phone* OR mobile telephone* OR cell phone* OR cellular phone* OR cellular telephone* OR electronic media* OR portable media device* OR tablet phone* OR tablet adj3 device).tw,kw. |
|  | 31 | (bedroom or bed or room or household or house).tw,kw. |
| Movement | 32 | Sedentary Lifestyle/ |
|  | 33 | (sedentary or inactiv* or (lack adj2 activity)).tw,kw. |
|  | 34 | ((low adj3 energy expend*) or physical* inactiv*).tw,kw. |
|  | 35 | ((chair or stroller or car or automobile* or auto or motor vehicle* or bus or indoor* or in-door or computer) adj3 time).tw,kw. |
|  | 36 | sitting.tw,kw. |
|  | 37 | Physical Activity.mp. |
|  | 38 | exp Exercise/ |
|  | 39 | exp Exercise Movement Techniques/ |
|  | 40 | exp Exercise Therapy/ |
|  | 41 | Physical Exertion/ |
|  | 42 | exp "Physical Education and Training"/ |
|  | 43 | exp Sports/ |
|  | 44 | (sport$ or bicycl$ or swim$ or walk$ or run$ or jog$).tw,kf. |
|  | 45 | (physical$ adj2 activ$).tw,kf. |
|  | 46 | (aerobic adj2 (train$ or active$)).tw,kf. |
|  | 47 | "Play and Playthings"/ and (activ* or outdoor*).tw,kf. |
|  | 48 | ((activ* or outdoor*) adj3 play*).tw,kf. |
|  | 49 | playground*.tw,kf. |
|  | 50 | active.ti. and (space* or behavio?r* or transport* or commut* or neighbo?rhood* or park* or game* or gaming or lifestyle).mp. |
|  | 51 | (active adj3 (space* or behavio?r* or transport* or commut* or neighbo?rhood* or park* or game* or gaming or lifestyle)).tw,kf. |
|  | 52 | prone position*.mp. or floor time.tw,kf. |
|  | 53 | *Or/24-31* |
|  | 54 | *23 and 53* |
|  | 55 | *Or/32-52* |
|  | 56 | *23 and 55* |
|  | 57 | *56 not (cerebral palsy or asthma or cystic fibrosis or autism).*tw,kf,mp. |

# Figure S1. Flowchart outlining identification of papers for inclusion

Additional papers identified through reference search
n = 1

Full text articles included in review
n = 31^*^^

- Movement behaviors n= 12
- Screen-based studies n = 23

** 4 articles contributed to both movement behaviors and screen-based studies*

*^ 29 unique study cohorts; 2 articles reported data from the same study*

Records identified up to March 2019

(after de-duplication)

n = 1604

MEDLINE: n = 647 EMBASE: n = 259

CINAHL: n = 132 PsycINFO: n = 349

CENTRAL: n = 194 Web of Science n = 167

Papers excluded based on title and abstract

n = 1514

Papers excluded based on full text

n = 50

- Inappropriate study population n = 12
- Inappropriate outcome measure n = 5
- No association described n = 17
- Other reason n = 16

Full texts retrieved and read in full

n = 90

Title and abstract screened

n = 1604

n=37704

# Table S1. Quality assessment individual studies

| Study author and year | Selection bias^a^ | Performance bias^b^ | Detection bias^c^ | Attrition bias^d^ | Selective reporting bias^e^ | Other bias^f^ |
| --- | --- | --- | --- | --- | --- | --- |
| Ahn et al. 2016 [43] | Unclear | Unclear | Unclear | Unclear | High | Low |
| Cespedes et al. 2014 [17] | Unclear | Unclear | High | High | Low | Low |
| Chonchaiya et al. 2017 [30] | High | High | High | Unclear | High | High |
| De Bock et al. 2013 [36] | Unclear | High | High | Low | Unclear | Low |
| Duraccio et al. 2017 [31] | High | Unclear | Unclear | Low | Unclear | Unclear |
| Garrison et al. 2011 [32] | High | High | Unclear | Unclear | Unclear | Unclear |
| Genuneit et al. 2018 [37] | Unclear | High | Unclear | High | High | High |
| Hager et al. 2016 [33] | High | Low | Unclear | High | Unclear | Low |
| Hauck et al. 2018 [26] | High | Unclear | Unclear | Unclear | Low | Low |
| Ikeda et al. 2012 [29] | Unclear | High | High | Unclear | Low | Low |
| Iwata et al. 2011 [44] | High | High | Low | Low | Unclear | Low |
| Krejci et al. 2011 [46] | Unclear | High | High | High | Unclear | Low |
| Magee et al. 2014 [50] | High | High | High | High | Unclear | Low |
| Marinelli et al. 2014 [38] | Low | High | High | High | Low | Low |
| McDonald et al. 2014 [39] | Low | High | Unclear | High | Low | Low |
| Ji et al. 2018 [45] | low | Unclear | Low | Unclear | High | Low |
| Mindell et al. 2013 [53] | High | High | Unclear | Unclear | Low | High |
| Nathanson et al. 2018 [34] | High | High | Low | Unclear | Low | Low |
| Nathanson et al. 2014 [35] | Unclear | High | High | Unclear | Low | Low |
| Nevarez et al. 2010 [18] | Low | High | High | High | High | Low |
| Ota et al. 2007 [48] | High | High | High | Low | Low | Low |
| Plancoulaine et al. 2015 [38] | Unclear | High | High | High | Unclear | Low |
| Reynaud et al. 2016 [39] | Unclear | High | High | High | Low | Low |
| Séguin et al. 2016 [28] | High | Unclear | Low | Low | High | Low |
| Sijtsma et al. 2015 [42] | Low | High | High | Unclear | Low | High |
| Taylor et al. 2015 [21] | Unclear | Low | Low | Unclear | Low | Low |
| Vijakkhana et al. 2015 [47] | High | High | High | Low | Low | High |
| Wang et al. 2019 [49] | Low | High | Low | Low | Low | Low |
| Williams et al. 2014 [20] | Unclear | Low | Low | Unclear | Low | Low |
| Xu H et al. 2016 [51] | High | High | Unclear | Unclear | Low | Low |
| Zhang et al. 2019 [52] | High | High | Low | High | Low | Low |

^a^ Risk classed as high if sample is convenience sample; risk classed as unclear if generalisability of sample is unknown

^b^ Risk classed as high if exposure measure is not validated; risk classed as unclear if exposure measure appears to be validated but psychometric properties are not reported

^c^ Risk classed as high if outcome measure is not validated; risk classed as unclear if outcome measure appears to be validated but psychometric properties are not reported

^d^ Risk classed as high if large amount of data is missing and reasons are not explained; risk classed as unclear if no information on missing data was provided or participants characteristics of missing data were not provided

^e^ Risk classed as high if pre-specified outcomes were missing from the results; risk classed as unclear if questions were raised about how final models were decided on

^f^ Risk classed as high if authors failed to control for confounders; risk classed as unclear if questions were raised about statistical methods used
